# Improving clinical efficiency using retrieval‐augmented generation in urologic oncology: A guideline‐enhanced artificial intelligence approach

**DOI:** 10.1002/bco2.427

**Published:** 2024-12-10

**Authors:** Harry Collin, Matthew J. Roberts, Kandice Keogh, Amila Siriwardana, Marnique Basto

**Affiliations:** ^1^ Department of Urology Royal Brisbane and Women's Hospital Herston Queensland Australia; ^2^ Faculty of Medicine The University of Queensland Brisbane Queensland Australia; ^3^ Faculty of Medicine University of Queensland Centre for Clinical Research Brisbane Queensland Australia

**Keywords:** artifical intelligence, cancer, chatgpt, European Association of Urology, renal cell carcinoma, retrieval‐augmented generation, urology

## 1 INTRODUCTION

Artificial intelligence (AI) in urology is evolving and has rapidly expanded since the release of ChatGPT and other large language models (LLMs). Early studies have found that AI‐generated patient information is moderate to high quality for patient questions across multiple uro‐oncology domains.^1^ Extension into clinical decision‐making suggests that ChatGPT can make decisions aligned with evidence‐based medicine.^2^

The key limitation of the publicly available ChatGPT (version 3.5) has been a reliance on knowledge confined to data published prior to September 2021.^3^ Subscription‐based ChatGPT Plus has since released ChatGPT 4.0, which is capable of web browsing. Equipped with the European Association of Urology (EAU) Guidelines, responses to urological queries are of higher quality,^4^ and ChatGPT 4.0 has been shown to make complex medical decisions concordant with those discussed in multidisciplinary team meetings.^5^ Furthermore, ChatGPT 4.0 allows LLMs to be curated for a specific task with retrieval‐augmented generation (RAG), whereby a Generative Pre‐trained Transformer (GPT) can use additional context provided by specialised materials to improve the accuracy of responses. Such advancements may realise the potential of AI systems to further urology practice via incorporation of up‐to‐date and highly specialised knowledge.

For instance, post‐treatment imaging surveillance for renal cell carcinoma (RCC) is a common yet challenging clinical scenario due to disconcordance between histopathological diversity and guideline algorithms. The EAU Guidelines offer structured recommendations for follow‐up imaging surveillance, which is pivotal for timely detection of cancer recurrence. However, effective clinical application of these guidelines requires specialised understanding of histopathology and can often be repetitive and time‐consuming particularly when assigned to junior doctors.

This study aimed to test a GPT customised with RAG to interpret post‐nephrectomy RCC histopathology reports and determine recommended follow‐up surveillance imaging according to EAU Guidelines.

A RAG system was created using ChatGPT 4.0 (OpenAI via ChatGPT Plus, https://chat.openai.com/gpts/editor). The 2023 EAU Guidelines on RCC were uploaded to the GPT including Chapter 3 (Epidemiology, Aetiology and Pathology), Chapter 4 (Staging and Classification Systems) and Chapter 8 (Follow‐Up in RCC). Code Interpreter capabilities were enabled, which allows the GPT to retrieve uploaded files and analyse data. Web browsing was disabled. No formal coding training or experience was required.

All instructions for the GPT were in free text (shown in Table S1). Instructions were written as three steps: interpret histopathology, determine surveillance regimen and output. The GPT was provided with clear directives to determine the risk profile (low, intermediate, or high risk) according to the EAU Guidelines, which uses Leibovich score for clear cell RCC (ccRCC) or histopathological stage and grade for non‐ccRCC. The GPT was then instructed to recommend a follow‐up imaging surveillance regimen, based on a template relative to risk profile (EAU Guidelines, tab. 8.1).

Simulated histopathology reports were created to represent all possible risk profiles (low, intermediate and high) relevant to the EAU Guidelines for the most common RCC subtypes—ccRCC, papillary (pRCC) and chromophobe (chRCC) tumours. Reports were structured according to International Society of Urological Pathology (ISUP) guidelines. Per the NHMRC National Statement on Ethical Conduct in Human Research 2023, this study did not require ethics committee approval as it utilised theoretical cases and does not meet the definition of human or animal research. Each report was input to the custom GPT on 15 January 2024. Responses were reviewed by two board‐certified urologists for concordance with the EAU Guidelines.

**FIGURE 1:**
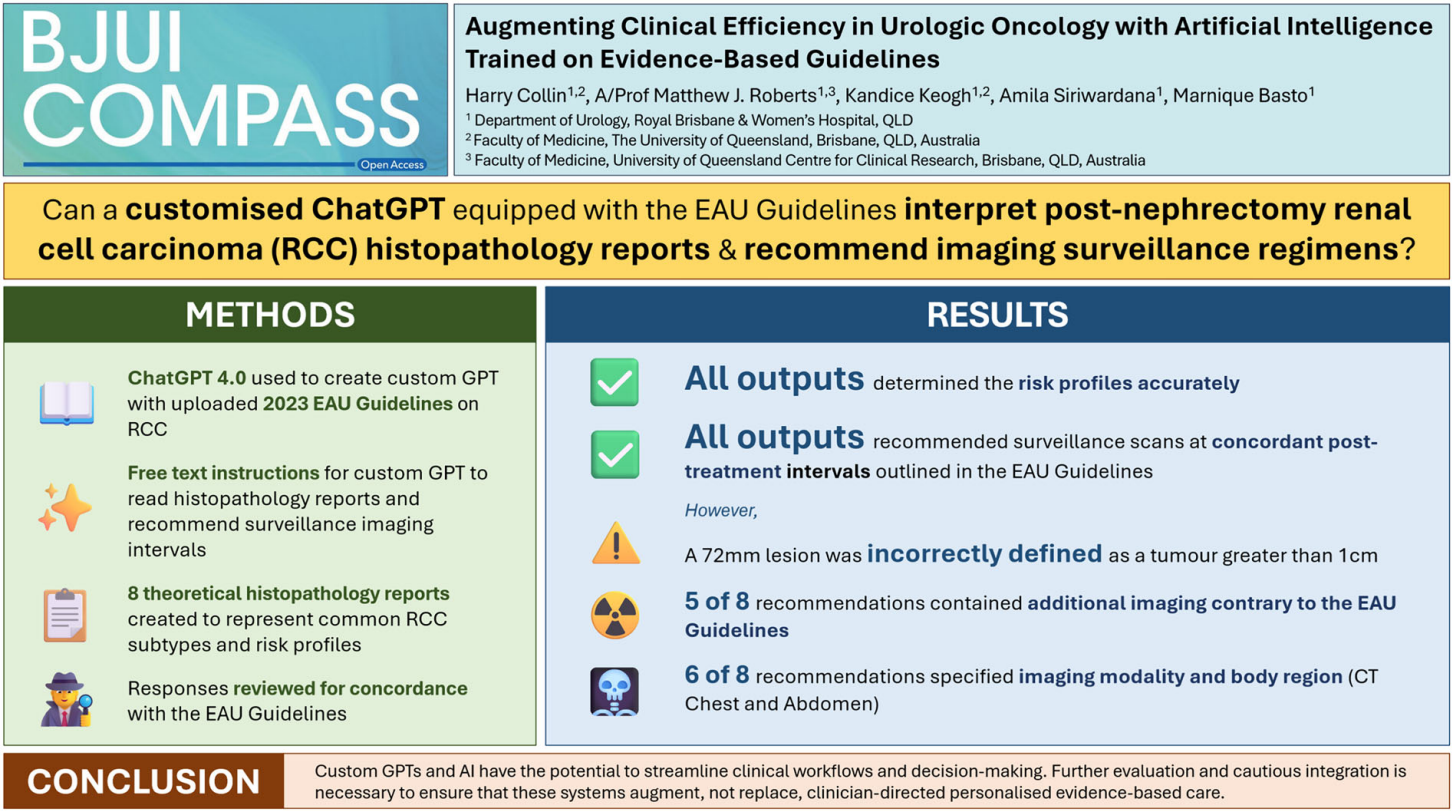
Augmenting clinical efficiency in urologic oncology with artificial intelligence trained on evidence‐based guidelines.

Full histopathology reports and their raw outputs are shown in Table S2. Results are summarised in Figure 1, and concordance of custom GPT outputs with the EAU Guidelines for each simulated histopathology report is shown in Figure S3.

The custom GPT correctly determined all RCC risk profiles. All but one Leibovich score was correct (a 72 mm lesion was incorrectly defined as a tumour greater than 1 cm). All outputs recommended surveillance scans at the post‐treatment intervals outlined in the EAU Guidelines. Three of the eight surveillance regimens (38%) were precisely concordant, while the remaining five contained additional imaging (3‐month imaging for intermediate risk and 30‐month imaging for high risk). The custom GPT proposed 2‐yearly scans beyond 5 years for low‐risk pRCC, contrary to the EAU Guidelines that suggest no further surveillance.

Most (6/8) recommendations specified imaging modality and body region (CT Chest and Abdomen). All outputs stated a guideline basis for the recommendation. Some outputs recommended renal and cardiovascular monitoring, which is mentioned in Chapter 8 of the EAU Guidelines, despite no specific prompting.

This study demonstrates the initial potential of RAG AI systems with integrated clinical guidelines to interpret results and make recommendations. This novel approach indicates the capacity of custom GPTs to handle complex and algorithmic tasks, which are often time‐consuming and prone to human error. The surveillance regimens (38% concordance) generated under tailored instructions may show improvement over non‐specialised ChatGPT 4 outputs, which lack focused access to specific guidelines and resulted in only 26% guideline concordance for prostate cancer.^6^ Our results also compare favourably to studies using web‐enabled ChatGPT 4.0 where 27% of responses to questions on kidney cancer, adapted from the EAU Guidelines, were of excellent quality.^7^ Surveillance regimens were consistently safe, with no missing interval scans and additional scans in 62% of regimens, indicating a cautious approach.

AI supplementation of specialised urology knowledge has previously required programming skills.^4^, ^8^ In this novel approach with a RAG design, we showed accurate, safe outputs from free text instructions without prior coding training. Consequently, ChatGPT 4.0 enables medical professionals to combine highly specialised knowledge with AI to enhance their clinical practice to their needs.

The inevitable introduction of AI to clinical settings must be met with close oversight from clinicians, especially when nuanced clinical judgements are involved. Here, the custom GPT miscalculated the Leibovich score from one theoretical report and could not consistently translate risk profile into a precisely concordant surveillance regimen. Conversely, unprompted recommendations promoted individualised care, such as renal and cardiovascular monitoring, so insights into comprehensive interpretation to enhance clinical practice were present.

A limitation of this study was partial inclusion of a single international guideline, which, despite being endorsed by 75 international societies, may limit GPT comprehensive integration. Future studies and AI developments could consider other guidelines (e.g., American Urological Association, National Institute for Health and Care Excellence). Ideally, development of a multi‐guideline framework that utilises a decision‐tree methodology could harness the power of AI to select and synthesise the most pertinent guidelines relevant to the local jurisdiction (limiting conflicts) or patient preferences for individualised care. Furthermore, web browsing was disabled to focus the AI on the uploaded guidelines but may have limited wider information access and integration, potentially affecting its handling of complex queries. Additionally, while the small series of histopathology report aimed to mitigate the custom GPT analysis complexity, future expansion of source number and content, as well as a training set, may further assess and enhance the custom GPT capabilities.

In conclusion, this focused evaluation of a GPT with integrated clinical guidelines illustrated the potential of AI, particularly RAG systems, for decision‐making accuracy across the most common histopathological subtypes. Future incorporation may streamline clinical workflows and decision‐making, but only with further evaluation, and cautious integration to ensure that these systems augment, not replace, clinician‐directed personalised evidence‐based care.

## CONFLICT OF INTEREST STATEMENT

There are no conflicts of interest.

## Supporting information


**Figure S1.** Custom GPT Instructions. The custom GPT is available at https://chat.openai.com/g/g-eIATeIq2o-rcc-surveillance-imaging.
**Table S2**. Input (histopathology report) to custom GPT with respective output.
**Figure S3.** Concordance of ChatGPT‐determined risk profiles and surveillance regimens to the EAU Guidelines on RCC.

## References

[1] Musheyev D , Pan A , Loeb S , Kabarriti AE . How well do artificial intelligence Chatbots respond to the top search queries about urological malignancies? Eur Urol. 2024;85(1):13–16. 10.1016/j.eururo.2023.07.004 37567827

[2] Zhou Z , Wang X , Li X , Liao L . Is ChatGPT an evidence‐based doctor? Eur Urol. 2023;84(3):355–356. 10.1016/j.eururo.2023.03.037 37061445

[3] OpenAI . GPT‐4 technical report [Internet]. arXiv; 2023 [cited 2023 Jul 30]. Available from: http://arxiv.org/abs/2303.08774

[4] Manolitsis I , Feretzakis G , Tzelves L , Kalles D , Katsimperis S , Angelopoulos P , et al. Training ChatGPT models in assisting urologists in daily practice. Stud Health Technol Inform. 2023;29(305):576–579. 10.3233/SHTI230562 37387096

[5] Gabriel J , Gabriel A , Shafik L , Alanbuki A , Larner T . Artificial intelligence in the urology multidisciplinary team meeting: can ChatGPT suggest European Association of Urology guideline‐recommended prostate cancer treatments? BJU Int. 2024;133(4):407–409. 10.1111/bju.16240 38009391

[6] Lombardo R , Gallo G , Stira J , Turchi B , Santoro G , Riolo S , et al. Quality of information and appropriateness of Open AI outputs for prostate cancer. Prostate Cancer Prostatic Dis. 2024;16:1–3. 10.1038/s41391-024-00789-0 38228809

[7] Ozgor F , Caglar U , Halis A , Cakir H , Aksu UC , Ayranci A , et al. Urological cancers and ChatGPT: assessing the quality of information and possible risks for patients. Clinical Genitourinary Cancer. 2024;22(2):454–457. 10.1016/j.clgc.2023.12.017 38246831

[8] Huang H , Lim FXY , Gu GT , Han MJ , Fang AHS , Chia EHS , et al. Natural language processing in urology: automated extraction of clinical information from histopathology reports of uro‐oncology procedures. Heliyon. 2023;9(4):e14793. 10.1016/j.heliyon.2023.e14793 37025805 PMC10070081

