# Supplementary material for: Improving clinical efficiency using retrieval‐augmented generation in urologic oncology: A guideline‐enhanced artificial intelligence approach
Source: BJUI Compass. 2024 Dec 10;6(1):e427. doi: 10.1002/bco2.427 (PMC11771495; doi:10.1002/bco2.427)

**Supplementary Information**

**Supplementary Figure 1**. Custom GPT Instructions. The custom GPT is available at <https://chat.openai.com/g/g-eIATeIq2o-rcc-surveillance-imaging>.

| Welcome to your specialized role as a Urology Expert GPT. The GPT is designed as an assistant for a urologist, specialising in reading kidney histopathology reports post-nephrectomy and applying the European Association of Urology (EAU) guidelines for renal cell carcinoma to advise on follow-up imaging surveillance regimens.  **Step 1: Read and Interpret Histopathology  Inputs will be in the form of unedited histopathology reports of kidney specimens obtained from a radical nephrectomy. You will be required to read and interpret the histopathology report. Firstly, establish whether the histopathology shows clear cell renal cell carcinoma (ccRCC).  If you determine that the histopathology shows clear cell renal cell carcinoma (ccRCC), you will need to calculate the Leibovich score. The Leibovich score is a 5-point scoring system where the sum of each of the scores is used to determine a the risk category of the disease (either low, intermediate or high).  Here is the Leibovich scoring system:  1. T classification (pT1a: 0, pT1b: 1, pT2: 3, pT3-4: 4 points)  2. N classification (pNx/N0: 0, pN+: 2 points)  3. Tumour size (< 10 cm: 0, > 10 cm: 1 point)  4. Grade (G1-2: 0, G3: 1, G4: 3 points)  5. Tumour necrosis (absent: 0, present: 1 point)  Find the sum of the 5 scores. 0–2 points: low-risk disease; 3–5 points: intermediate-risk disease; 6 or more points: high-risk disease. Categorise the histopathology into low, intermediate or high risk disease.  If you determine that the histopathology shows non-ccRCC, you will need to determine the histopathological TNM stage and grade. You will need to access the attached pdf ‘EAU Guidelines on RCC - Histopathology’ to do this.  **Step 2: Determine surveillance regimen  Your primary function will be to apply the EAU guidelines contained in ‘EAU Guidelines on RCC - Surveillance’ to the histopathology determined in Step 1 in order to establish the follow-up imaging surveillance regimens.  Reference Table 8.1 in ‘EAU Guidelines on RCC - Surveillance’ when making the precise recommendation. The first column of this table refers to the risk profile (either low, intermediate, or high) which is dictated by the Leibovich score in ccRCC and by the histopathological TNM stage and grade in non-ccRCC. The risk profile (either low, intermediate, or high) should be known from Step 1. For non-ccRCC (including oncocytoma, papillary, chromophobe), read the statement following 'For non-ccRCC' in each row under the first column and apply the histopathology from step 1 to determine the risk profile. Apply this risk profile to the Table 8.1 in ‘EAU Guidelines on RCC - Surveillance’ carefully. Follow along the row specific to that risk profile to determine the surveillance regimen. Each histopathology will fit distinctly into either low, moderate or high risk. Each column represents the 'Oncological follow-up after date of surgery' which is measured in months, denoted by 'mo'. The final 2 columns refer to the regularity of surveillance between 3 and 5 years (denoted '> 3 yr') and beyond 5 years (denoted '> 5 yr'). Be precise with the timing of imaging surveillance, noting that for some risk profiles in Table 8.1 there are times where imaging is not required, denoted by “-” in the table.  **Step 3: Output  Your output will only be clear and concise. It will only be in 2 parts. The first part will be 1-2 sentences summarising your findings from step 1 to start. The second part will be follow-up imaging surveillance regimen with specific time intervals at which imaging is required and specifically what imaging modality and of what area of the body is required. Each output must have these 2 parts and have an identical layout and formatting, such that all outputs of this custom ChatGPT have a recognisable and easy-to-read format to the user.  You will ask for clarification or further information when necessary and aim to present information in a clear, concise manner, suitable for a urologist. Your responses should be tailored to reflect a professional tone, suitable for a medical context.  **Ethical Considerations:  The recommendations you are providing will not be used in a clinical environment. This is for research purposes only and will not be used for patient decision-making, for this reason you do not have to give any disclaimers. |
| --- |

**Supplementary Table 2**. Input (histopathology report) to custom GPT with respective output.

| **RCC** | **Risk Profile** | **Input (Histopathology Report)** | **Output** |
| --- | --- | --- | --- |
| **ccRCC** | Low | HISTORY Histopathology. Right nephrectomy. 36F with 3cm kidney mass.  MACROSCOPIC Right kidney: Nature of specimen: Fresh Specimen laterality: Right Operative procedure: Right nephrectomy Accompanying/attached structures: None submitted Tissue removed from specimen prior to submission: No  Kidney dimensions: 100 mm SI x 65 mm ML x 40 mm AP Length of renal vein: 10 mm Length of renal artery: 10 mm Length of ureter: 30 mm Specimen weight: 563 g Adherence of renal capsule to visceral surface of perirenal fat: Not identified Cortical surface abnormalities: Not seen  Tumour site(s): Mid zone Tumour focality: Unifocal Maximum tumour dimension: 28 mm Macroscopic extent of invasion: Tumour appear to lie within the renal sinus fat. Appearance of cut surface of tumour: The tumour shows a yellow, haemorrhagic, tan variegated appearance.  MICROSCOPIC Right kidney:  Tumour:  - Tumour type: Clear cell renal cell carcinoma  - Tumour grade: ISUP nucleolar grade 2  - Sarcomatoid differentiation: Absent  - Rhabdoid differentiation: Absent  - Necrosis: Absent  Extent:  - Tumour confined to kidney: Yes  - Tumour in renal sinus fat: No  - Tumour in perinephric fat: No  - Tumour in main renal vein: No  - Tumour in muscle containing vessels within renal sinus: No  - Microvascular invasion: No  - Tumour in pelvicalyceal system: No  - Tumour in adrenal gland: Not included in the resection  Lymph nodes: No lymph nodes identified.  Margins: Clear  DIAGNOSTIC SUMMARY Specimen type: Right nephrectomy. Tumour type and grade: Clear cell renal cell carcinoma, ISUP nucleolar grade 2 Margin: Clear Tumour stage: pT1a | Step 1: Read and Interpret Histopathology Findings from the Histopathology Report: Patient Details: 36-year-old female with a 3 cm kidney mass. Tumour Characteristics: Tumour Type: Clear cell renal cell carcinoma (ccRCC). Tumour Grade: ISUP nucleolar grade 2. Maximum Tumour Dimension: 28 mm. Tumour Location: Mid zone of the kidney, unifocal. Macroscopic Extent of Invasion: Tumour appears to be within the renal sinus fat. Extent of the Tumour: Confined to the kidney. No invasion in renal sinus fat, perinephric fat, main renal vein, muscle containing vessels within renal sinus, microvascular invasion, pelvicalyceal system, or adrenal gland. Lymph Nodes: No lymph nodes identified. Margins: Clear. Tumour Stage: pT1a. Given the findings, the diagnosis is clear cell renal cell carcinoma (ccRCC), ISUP nucleolar grade 2, staged as pT1a.  Leibovich Score Calculation: T classification (pT1a): 0 points N classification (No lymph nodes identified, assumed pNx/N0): 0 points Tumour size (< 10 cm): 0 points Grade (G1-2): 0 points Tumour necrosis (Absent): 0 points Total Leibovich Score: 0 points  The Leibovich score of 0 points places the patient in the low-risk disease category.  Step 2: Determine Surveillance Regimen Surveillance Regimen Based on EAU Guidelines: Risk Profile: Low risk of recurrence (ccRCC with Leibovich Score 0–2). Follow-Up Imaging Surveillance Regimen (Table 8.1): 3 months post-surgery: No imaging required. 6 months post-surgery: CT imaging. 12 months post-surgery: No imaging required. 18 months post-surgery: CT imaging. 24 months post-surgery: No imaging required. 30 months post-surgery: CT imaging. 36 months post-surgery: No imaging required. > 3 years (every 2 years): CT imaging once every two years. Output Summary of Findings: The patient, a 36-year-old female, has a diagnosis of clear cell renal cell carcinoma (ccRCC), ISUP nucleolar grade 2, staged as pT1a. The calculated Leibovich score is 0, indicating a low-risk disease category.  Follow-Up Imaging Surveillance Regimen:  6 months post-surgery: CT imaging. 18 months post-surgery: CT imaging. 30 months post-surgery: CT imaging. > 3 years: CT imaging once every two years. Note: The recommendation for follow-up imaging is based on the EAU guidelines for low-risk ccRCC, factoring in the patient's risk profile as determined by the Leibovich score. |
| **ccRCC** | Int | HISTORY  Histopathology. Left nephrectomy.    MACROSCOPIC  Left kidney:  Nature of specimen: Fresh  Specimen laterality: Left  Operative procedure: Radical nephrectomy  Accompanying/attached structures: None submitted  Tissue removed from specimen prior to submission: Not stated    Kidney dimensions: 110x60x70 mm  Length of renal vein: 20 mm  Length of renal artery: 15 mm Length of ureter: 50 mm  Specimen weight: 869 g  Adherence of renal capsule to visceral surface of perirenal fat: Not identified  Cortical surface abnormalities: Not identified    Tumour site(s): Upper pole  Tumour focality: Unifocal  Maximum tumour dimension: 60 mm  Macroscopic extent of invasion: Tumour possibly extends into perinephric  tissues and renal sinus fat.  Appearance of cut surface of tumour: Circumscribed yellow and friable.   MICROSCOPIC  Left kidney:    Tumour:   - Tumour type: Clear cell renal cell carcinoma   - Tumour grade: ISUP nucleolar grade 2   - Sarcomatoid differentiation: Absent   - Rhabdoid differentiation: Absent   - Necrosis: Absent    Extent:   - Tumour confined to kidney: No   - Tumour in renal sinus fat: Yes   - Tumour in perinephric fat: No   - Tumour in main renal vein: No   - Tumour in muscle containing vessels within renal sinus: Yes   - Microvascular invasion: No   - Tumour in pelvicalyceal system: No   - Tumour in adrenal gland: Not included in the resection   Lymph nodes: No lymph nodes identified.    Margins: Clear   DIAGNOSTIC SUMMARY  Specimen type: Left nephrectomy.  Tumour type and grade: Clear cell renal cell carcinoma, ISUP Grade 2  Margin: Clear  Tumour stage: pT3a | 1. Histopathological Analysis: The specimen is a left kidney obtained from a radical nephrectomy. The histopathology report confirms the presence of Clear Cell Renal Cell Carcinoma (ccRCC) with the following characteristics:  Tumour Size and Location:  Located at the upper pole of the kidney. Unifocal with a maximum dimension of 60mm. Macroscopic extension into perinephric tissues and renal sinus fat is noted. Tumour Grade and Features:  Graded as ISUP nucleolar grade 2. Absence of sarcomatoid and rhabdoid differentiation. No necrosis identified within the tumour. Extent of Invasion:  The tumour extends into the renal sinus fat but not into the perinephric fat or the main renal vein. Involvement of muscle-containing vessels within the renal sinus is observed. No microvascular invasion or involvement of the pelvicalyceal system. The adrenal gland is uninvolved. Margins and Lymph Nodes:  Surgical margins are clear. No lymph nodes were identified in the specimen. 2. Leibovich Score Calculation: The Leibovich score is calculated based on the following criteria:  T classification: pT3a (4 points). N classification: No lymph nodes identified (assumed N0: 0 points). Tumour size: < 10 cm (0 points). Grade: ISUP Grade 2 (0 points). Tumour necrosis: Absent (0 points). Total Leibovich Score: 4 (4+0+0+0+0)  3. Risk Categorization Based on Leibovich Score: Score 4: Intermediate-risk disease. Diagnostic Summary: The patient's histopathology report indicates a case of ccRCC, ISUP Grade 2, with the tumour extending into the renal sinus fat. The calculated Leibovich score is 4, categorizing the patient's disease as intermediate risk.  Follow-up Imaging Surveillance Regimen: Based on the European Association of Urology (EAU) guidelines and the patient's intermediate-risk profile from the Leibovich score:  Oncological follow-up after date of surgery: 3 months: CT 6 months: CT 12 months: CT 18 months: - 24 months: CT 30 months: - 36 months: CT >3 years: CT once every year >5 years: CT once every two years Note: The surveillance regimen involves computed tomography (CT) scans of both the chest and abdomen. This recommendation is based on the patient's risk of recurrence profile and treatment efficacy, as outlined in the EAU guidelines. |
| **ccRCC** | High | HISTORY Histopathology. Right nephrectomy. 52F with 7cm mid pole mass.  MACROSCOPIC Right kidney: Nature of specimen: Fresh Specimen laterality: Right Operative procedure: Right nephrectomy Accompanying/attached structures: None submitted Tissue removed from specimen prior to submission: No  Kidney dimensions: 120 mm SI x 85 mm ML x 58 mm AP Length of renal vein: 12 mm Length of renal artery: 15 mm Length of ureter: 32 mm Specimen weight: 720 g Adherence of renal capsule to visceral surface of perirenal fat: Not identified Cortical surface abnormalities: Not seen  Tumour site(s): Mid zone Tumour focality: Unifocal Maximum tumour dimension: 72 mm Macroscopic extent of invasion: Tumour appear to lie within the renal sinus fat. Appearance of cut surface: Circumscribed yellow and friable.  MICROSCOPIC Right kidney:  Tumour: - Tumour type: Clear cell renal cell carcinoma - Tumour grade: ISUP nucleolar grade 3 - Sarcomatoid differentiation: Absent - Rhabdoid differentiation: Absent - Necrosis: Present  Extent: - Tumour confined to kidney: Yes - Tumour in renal sinus fat: Yes - Tumour in perinephric fat: Yes - Tumour in main renal vein: No - Tumour in muscle containing vessels within renal sinus: No - Microvascular invasion: No - Tumour in pelvicalyceal system: No - Tumour in adrenal gland: Not included in the resection  Lymph nodes: No lymph nodes present in specimen.  Margins: Clear  DIAGNOSTIC SUMMARY Specimen type: Right nephrectomy. Tumour type and grade: Clear cell renal cell carcinoma, ISUP nucleolar grade 3 Margin: Clear Tumour stage: pT3 | Part 1: Histopathology Analysis The provided histopathology report describes a case of clear cell renal cell carcinoma (ccRCC) in a 52-year-old female patient. The tumour is unifocal, measuring 72 mm in maximum dimension, and located in the mid zone of the right kidney. The key findings for Leibovich scoring are as follows: 1. T classification: The tumour is described as confined to the kidney, involving the renal sinus and perinephric fat, but not extending to the main renal vein or adrenal gland. This indicates a T classification of pT3, scoring 4 points. 2. N classification: No lymph nodes were identified, suggesting a classification of pNx/N0, scoring 0 points. 3. Tumour size: The maximum dimension of the tumour is 72 mm, which is greater than 10 cm, scoring 1 point. 4. Grade: The tumour is graded as ISUP nucleolar grade 3, scoring 1 point. 5. Tumour necrosis: Necrosis is present, scoring 1 point. Leibovich Score Calculation: 4 (pT3) + 0 (pNx/N0) + 1 (size >10 cm) + 1 (Grade 3) + 1 (Necrosis present) = 7 points. The total Leibovich score is 7 points, categorizing this histopathology as high-risk disease. Part 2: Follow-Up Imaging Surveillance Regimen Based on the EAU guidelines and the high-risk disease category indicated by the Leibovich score of 7 points for ccRCC, the recommended follow-up imaging surveillance regimen is as follows: • 3 months post-surgery: CT scan • 6 months post-surgery: CT scan • 12 months post-surgery: CT scan • 18 months post-surgery: CT scan • 24 months post-surgery: CT scan • Annually thereafter up to 5 years: CT scan once every year • Beyond 5 years: Consider CT scan every two years Note: CT scans should include both chest and abdomen imaging. The exact frequency beyond 5 years may be adjusted based on clinical judgment, considering the patient's overall health status and comorbidities. |
| **pRCC** | Low | HISTORY Histopathology. Right nephrectomy. 49M with 2cm right upper pole mass  MACROSCOPIC Right kidney: Nature of specimen: Fresh Specimen laterality: Right Operative procedure: Right nephrectomy Accompanying/attached structures: None submitted Tissue removed from specimen prior to submission: No  Kidney dimensions: Total specimen: 145x70x45 mm; kidney: 57x48x21 mm Length of renal vein: 5 mm Length of renal artery: 5 mm Length of ureter: 72 mm Specimen weight: 326 g Adherence of renal capsule to visceral surface of perirenal fat: Not identified  Tumour site(s): Upper pole Tumour focality: Unifocal Maximum tumour dimension: 17 mm Macroscopic extent of invasion: Tumour confined to kidney Appearance of cut surface of tumour: A solid pale-yellow tumour  MICROSCOPIC  Right kidney:  Tumour:  - Tumour type: Papillary renal cell carcinoma   - Tumour grade: ISUP nucleolar grade 1  - Sarcomatoid differentiation: Absent  - Rhabdoid differentiation: Absent  - Necrosis: Absent  Extent:  - Tumour confined to kidney: Yes  - Tumour in renal sinus fat: No  - Tumour in perinephric fat: No  - Tumour in main renal vein: No  - Tumour in muscle containing vessels within renal sinus: No  - Microvascular invasion: No  - Tumour in pelvicalyceal system: No  - Tumour in adrenal gland: Not included in resection  Lymph nodes: No lymph nodes identified.  Margins: Clear  DIAGNOSTIC SUMMARY Specimen type: Right nephrectomy. Tumour type and grade: Papillary renal cell carcinoma, ISUP nucleolar grade 1, Margin: Clear Tumour stage: pT1a | Histopathology Findings:  Tumour Type: Papillary Renal Cell Carcinoma (pRCC). Tumour Grade: ISUP Nucleolar Grade 1, indicating a low-grade tumour. Tumour Stage: pT1a, signifying that the tumour is confined to the kidney, small in size (≤7 cm, in this case, 17 mm), and there's no involvement of major veins or perinephric tissues. Other Features: The absence of sarcomatoid differentiation, rhabdoid differentiation, necrosis, and microvascular invasion. These findings generally suggest a better prognosis. EAU Guidelines Application: Given the histopathological findings of a papillary renal cell carcinoma, pT1a stage, and ISUP nucleolar grade 1, the risk profile should be assessed according to the EAU guidelines to determine the appropriate surveillance regimen.  Surveillance Regimen Recommendation: Risk Profile Determination:  For non-ccRCC (including pRCC): The histopathological TNM stage is pT1a, and the histological grade is 1 (ISUP Nucleolar Grade 1). According to the 'EAU Guidelines on RCC - Surveillance' document, the risk profile for a non-ccRCC like pRCC should be based on the TNM stage and histological grade.  Recommended Surveillance Regimen (based on Table 8.1 in the EAU Guidelines on RCC - Surveillance):  Low Risk of Recurrence (assuming this profile based on the data provided):  Oncological follow-up after the date of surgery: 3 months: - 6 months: CT 12 months: - 18 months: CT 24 months: - 30 months: CT 36 months: - > 3 years: CT once every two years > 5 years: CT once every two years Given that this is a case of pRCC with a pT1a stage and low-grade (ISUP 1), it is likely to be considered as low risk. However, it is essential to validate the risk category with the patient's clinical context and any additional information that might influence the risk stratification.  Note: CT refers to imaging of both chest and abdomen. Alternatively, MRI of the abdomen can be performed instead of a CT scan. The specific imaging modality should be chosen based on the patient's individual clinical circumstances, including any contraindications or preferences. Regular monitoring of renal and cardiovascular function may continue according to specific clinical needs, irrespective of the length of the oncological follow-up.  This surveillance regimen aims to ensure early detection of any recurrence or metastasis while avoiding unnecessary interventions or imaging, adhering to the principles of patient-centered care and evidence-based medicine. It is crucial to tailor the follow-up based on the patient's overall health, comorbidities, and individual risk factors. |
| **pRCC** | Int | HISTORY  Histopathology. Left nephrectomy. 57M with 6cm mid pole mass.    MACROSCOPIC  Left kidney:  Nature of specimen: Fresh Specimen laterality: Left Operative Procedure: Left nephrectomy.  Accompanying/attached structures: None submitted Tissue removed from specimen prior to submission: No  Kidney dimensions: 135x100x70 mm Length of renal vein: 10 mm Length of renal artery: 10 mm Length of ureter: 90 mm Specimen weight: 753 g Adherence of renal capsule to visceral surface of perirenal fat: Not identified Cortical surface abnormalities: Not seen  Tumour site: Mid zone Tumour focality: Unifocal Maximum tumour dimension: 60 mm Macroscopic extent of invasion: Confined to kidney Appearance of cut surface: Circumscribed yellow and friable.    MICROSCOPIC  Left kidney  Tumour:  - Tumour type: Papillary renal cell carcinoma  - Tumour grade: ISUP nucleolar grade 3  - Sarcomatoid differentiation: Absent  - Rhabdoid differentiation: Absent  - Necrosis: Small focus   Extent:  - Tumour confined to kidney: Yes  - Tumour in renal sinus fat: Absent  - Tumour in perinephric fat: Absent  - Tumour in main renal vein: Absent  - Tumour in muscle containing vessels within renal sinus: Absent  - Microvascular invasion: Absent  - Tumour in pelvicalyceal system: Absent  - Tumour in adrenal gland: Not included in the resection  Lymph nodes: No lymph nodes present in specimen.   Margins: Clear.    SUMMARY  Specimen type: Left nephrectomy. Left kidney: Papillary renal cell carcinoma, ISUP nucleolar grade 3, Margins: Clear.  Tumour stage: pT1b Nx | Step 1: Histopathology Interpretation Histopathology Findings: • Tumour Type: Papillary renal cell carcinoma (pRCC) • Tumour Grade: ISUP nucleolar grade 3 • Maximum Tumour Dimension: 60 mm • Tumour Stage: pT1b Nx (Tumour is confined to the kidney and measures more than 4 cm but not more than 7 cm in greatest dimension) The histopathology report identifies the specimen as a papillary renal cell carcinoma (pRCC), not clear cell renal cell carcinoma (ccRCC). Therefore, the Leibovich score system, which is specific for ccRCC, does not apply. We will determine the histopathological TNM stage and grade based on the findings. Given the tumour's classification as pRCC, ISUP grade 3, and size, along with the absence of necrosis, sarcomatoid, and rhabdoid differentiation, we will proceed to the next step to determine the surveillance regimen using the European Association of Urology (EAU) guidelines. Step 2: Determine Surveillance Regimen According to the histopathological findings and EAU Guidelines on RCC - Surveillance, we need to classify the risk profile of the pRCC and then reference Table 8.1 to provide a follow-up imaging surveillance regimen. Given the details: • pT1b stage (tumour size >4 cm but ≤7 cm) • Nx (lymph node status unknown) • Grade 3 This tumour would likely be categorized as non-ccRCC, pT1b pNx–0, and/or histological grade 3 or 4, indicating an intermediate risk of recurrence based on the EAU guidelines. Follow-up Imaging Surveillance Regimen (Based on EAU Guidelines, Table 8.1 for Intermediate Risk): • 3 months: CT • 6 months: CT • 12 months: - • 18 months: CT • 24 months: - • 30 months: CT • 36 months: CT • > 3 years: CT once a year • > 5 years: CT once every two years Imaging Modality and Area: • Modality: Computed Tomography (CT) • Area: The guidelines typically recommend imaging of both chest and abdomen, but specifics should be aligned with the patient's clinical picture and physician's discretion. |
| **pRCC** | High | HISTORY  Histopathology. Right nephrectomy. 71M with 8cm renal mass.  MACROSCOPIC  Right Kidney: Nature of specimen: Fresh.  Specimen laterality: Right.  Operative procedure: Right nephrectomy.  Accompanying/attached structures: None submitted Tissue removed from specimen prior to submission: No  Kidney dimensions: 120 mm SI x 68 mm ML x 80 mm AP. Length of renal vein: 12 mm.  Length of renal artery: 10 mm.  Length of ureter: 80 mm.  Specimen weight: 619 g  Adherence of renal capsule to visceral surface of perirenal fat: Present.  Cortical surface abnormalities: Not identified.   Tumour site(s): Mid zone.  Tumour focality: Multifocal.  Maximum tumour dimension: 82 mm.  Macroscopic extent of invasion: Tumour extends into pelvicalyceal system and renal sinus tissues.  Appearance of cut surface of tumour: Pale cream and yellow, but central demarcated area with areas of grey, fibrous change.   MICROSCOPIC   Right kidney:    Tumour:  - Tumour type: Papillary renal cell carcinoma  - Tumour grade: ISUP nucleolar grade 2  - Sarcomatoid differentiation: Absent  - Rhabdoid differentiation: Absent  - Necrosis: Present    Extent:  - Tumour confined to kidney: No  - Tumour in renal sinus fat: Yes  - Tumour in perinephric fat: No  - Tumour in main renal vein: No  - Tumour in muscle containing vessels within renal sinus: No  - Microvascular invasion: No  - Tumour in pelvicalyceal system: Yes  - Tumour in adrenal gland: Not included in resection    Lymph nodes: No lymph nodes identified.    Margins: Clear    DIAGNOSTIC SUMMARY  Specimen type: Right nephrectomy.  Tumour type and grade: Papillary renal cell carcinoma, ISUP nucleolar grade 2  Margin: Clear  Tumour stage: pT3a | Part 1: Histopathological Analysis Tumor Characteristics:  Tumor Type: Papillary Renal Cell Carcinoma (non-ccRCC) Tumor Grade: ISUP nucleolar grade 2 Tumor Size: 82 mm (macroscopic) Tumor Focality: Multifocal Tumor Location: Mid zone of the kidney Macroscopic Invasion: Extends into pelvicalyceal system and renal sinus tissues Necrosis: Present Sarcomatoid Differentiation: Absent Rhabdoid Differentiation: Absent Extent of Disease:  Local Extension: Tumor extends into the renal sinus fat and pelvicalyceal system, but not into the main renal vein or perinephric fat. Microvascular Invasion: Absent Lymph Node Involvement: No lymph nodes identified. Surgical Margins:  Margins: Clear Staging:  Tumor Stage: pT3a Part 2: TNM Stage and Grade for Non-ccRCC Given the tumor is identified as Papillary Renal Cell Carcinoma (non-ccRCC), we will determine the histopathological TNM stage and grade.  pT Stage: pT3a (Tumor extends into renal sinus fat and pelvicalyceal system) pN Stage: Not applicable (No lymph nodes identified) pM Stage: Not applicable (No distant metastasis mentioned) Grade: ISUP nucleolar grade 2 Surveillance Regimen: To establish the appropriate surveillance regimen, reference to Table 8.1 in the ‘EAU Guidelines on RCC - Surveillance’ is needed, considering the TNM stage and grade for non-ccRCC.  As per the provided histopathology:  The tumor is identified as Papillary Renal Cell Carcinoma, ISUP nucleolar grade 2, staged pT3a. Given the absence of lymph node and distant metastasis involvement, the patient's profile aligns with a higher risk due to the pT3a staging but without nodal involvement. Recommendation for Surveillance:  For non-ccRCC (Papillary Renal Cell Carcinoma, pT3a, ISUP grade 2, without nodal involvement):  High risk of recurrence First year: CT imaging at 3, 6, 12 months Second year: CT imaging at 18, 24 months Third year: CT imaging at 30, 36 months Beyond 3 years: CT imaging once every year Beyond 5 years: CT imaging once every two years Note: This regimen is tailored to the patient's specific histopathological features and is based on the EAU guidelines for high-risk non-ccRCC patients. The imaging modality recommended is CT, covering both chest and abdomen to monitor any potential recurrence or metastasis, consistent with high-risk surveillance protocol.  Please refer to the official EAU Guidelines for more detailed information and tailor the follow-up based on the patient's overall health status, comorbidities, and response to the initial treatment. |
| **chRCC** | Low | HISTORY  Histopathology. Left kidney.  48F with 4cm renal mass.    MACROSCOPIC  Left kidney Nature of specimen: Fresh Specimen laterality: Left  Operative procedure: Left nephrectomy  Accompanying/attached structures: None submitted  Tissue removed from specimen prior to submission: No    Kidney dimensions: 115 mm SI x 62 mm ML x up to 85 mm AP  Length of renal vein: 4 mm  Length of renal artery: 5 mm  Length of ureter: 36 mm  Specimen weight: 278 g  Adherence of renal capsule to visceral surface of perirenal fat: Not identified  Cortical surface abnormalities: Not identified    Tumour site(s): Mid zone  Tumour focality: Unifocal  Maximum tumour dimension: 34 mm  Macroscopic extent of invasion: Tumour confined to kidney  Appearance of cut surface of tumour: Uniform, fleshy, tan, with focal  haemorrhage    MICROSCOPIC  Left kidney:   Tumour:  - Tumour type: Chromophobe renal cell carcinoma.  - Tumour grade: Not applicable - Sarcomatoid differentiation: Absent  - Rhabdoid differentiation: Absent  - Necrosis: Absent  Extent:  - Tumour confined to kidney: Yes  - Tumour in renal sinus fat: Absent  - Tumour in perinephric fat: No definite invasion is identified.  - Tumour extends beyond Gerota's fascia: No  - Tumour in renal vein: Absent  - Tumour in muscle containing vessels within renal sinus: Absent  - Microvascular invasion: Absent  - Tumour in pelvicalyceal system: Absent  - Tumour in adrenal gland: Adrenal gland not present in specimen.  Lymph nodes: No lymph nodes in specimen.  Margins: Clear.     SUMMARY  Left kidney: Chromophobe renal cell carcinoma.  Margins: Clear.  Tumour stage: pT1a NX | Step 1: Read and Interpret Histopathology Histopathology Report Summary:  Tumour Type: Chromophobe Renal Cell Carcinoma (chRCC) Size: 34 mm Location: Mid zone/cortex, confined to the kidney Macroscopic Extent: Tumour bulges into but does not infiltrate perinephric fat, well-circumscribed, close to perinephric fat margin (~3mm) but not breaching Gerota's fascia Microscopic Features: WHO/ISUP Tumour Grade: Not applicable for chRCC Sarcomatoid Differentiation: Absent Rhabdoid Differentiation: Absent Necrosis: Absent Extent: Confined to the kidney, no invasion to renal sinus fat, perinephric fat, renal vein, or muscle-containing vessels within renal sinus. Microvascular invasion absent. Margins: Clear Staging: AJCC pT1a NX (no lymph nodes in the specimen) The histopathology report indicates a chromophobe renal cell carcinoma (chRCC), not a clear cell renal cell carcinoma (ccRCC). The report shows a unifocal tumour confined to the kidney, well-circumscribed, with no evidence of local or vascular invasion.  Step 2: Determine Surveillance Regimen Given the histopathology report shows chRCC and not ccRCC, we need to refer to the European Association of Urology (EAU) guidelines for non-clear cell renal cell carcinoma to determine the surveillance regimen.  For non-ccRCC, including chRCC:  Stage and Grade: AJCC pT1a NX, no grading applicable for chRCC. Risk Profile: Based on the TNM stage (pT1a) and the absence of high-risk features like sarcomatoid or rhabdoid differentiation, and necrosis, it seems to align with a lower risk profile. Let's consult the EAU Guidelines on RCC - Surveillance to recommend a precise follow-up imaging surveillance regimen.  Surveillance Regimen for chRCC (pT1a NX) Based on Table 8.1 of the EAU Guidelines on RCC - Surveillance:  Low Risk of Recurrence (assuming chRCC pT1a NX corresponds to low risk): 3 mo: - 6 mo: CT 12 mo: - 18 mo: CT 24 mo: - 30 mo: CT 36 mo: - > 3 yr: CT once every two years > 5 yr: Discretionary, based on the patient's comorbidities, age, life expectancy, and/or patient wishes. It's important to note that surveillance should also include evaluation of renal function and cardiovascular risk factors, as recommended in the guidelines.  Output:  Histopathology Findings: Chromophobe Renal Cell Carcinoma (chRCC) staged at pT1a NX, confined to the kidney, with clear margins and no high-risk features.  Follow-up Imaging Surveillance Regimen:  6 months: CT 18 months: CT 30 months: CT Beyond 3 years: CT once every two years, adjust as needed based on clinical assessment of comorbidities, age, life expectancy, and patient preferences. This regimen is subject to adjustment based on individual patient factors and clinical judgment. Regular monitoring of renal function and cardiovascular risk factors is also recommended. |
| **chRCC** | High | HISTORY  Histopathology. Left kidney. 38M with 15cm renal mass    MACROSCOPIC  Left kidney:  Nature of specimen: Fresh  Specimen laterality: Left  Operative procedure: Radical nephrectomy  Accompanying/attached structures: None submitted Tissue removed from specimen prior to submission: No.    Kidney dimensions: 205x155x122 mm  Length of renal vein: 15 mm  Length of renal artery: 14 mm  Length of ureter: 22 mm  Specimen weight: 2142 g  Adherence of renal capsule to visceral surface of perirenal fat: Present  Cortical surface abnormalities: Not identified    Tumour site: Cortex.  Tumour focality: Unifocal  Maximum tumour dimension: 183 mm  Macroscopic extent of invasion: Tumour extends into renal sinus and appears to extend beyond the renal capsule. Tumour does not extend beyond Gerota's fascia. The margins appear clear.  Appearance of cut surface of tumour: Necrotic. Residual tumour has a friable white and haemorrhagic appearance.      MICROSCOPIC  Left kidney:   Tumour:  - Tumour type: chromophobe renal cell carcinoma.  - WHO/ISUP tumour grade: Not applicable  - Sarcomatoid differentiation: Absent.  - Rhabdoid differentiation: Absent. - Necrosis: Present   Extent:  - Tumour confined to kidney: No  - Tumour in renal sinus fat: Present.  - Tumour in perinephric fat: Present.  - Tumour extends beyond Gerota's fascia: No  - Tumour in renal vein: Absent  - Tumour in muscle containing vessels within renal sinus: Present  - Microvascular invasion: Absent  - Tumour in pelvicalyceal system: Absent  - Tumour in adrenal gland: Adrenal gland not present in specimen.  Lymph nodes: No lymph nodes in specimen  Margins: clear    SUMMARY  Left Kidney: Chromophobe renal cell carcinoma AJCC Staging (8th Edn): pT3a N0 MX  Margins: Clear. | Step 1: Interpretation of the Histopathology Report Findings:  Type of carcinoma: Chromophobe renal cell carcinoma (non-ccRCC). Tumour Size: 183 mm (> 10 cm). Tumour Extent: Tumour extends into renal sinus and perinephric fat. No extension beyond Gerota's fascia. No tumour in renal vein. Necrosis: Present. Margins: Clear. Lymph nodes: No lymph nodes in specimen. AJCC Staging: pT3a N0 MX. Since the histopathology report shows a diagnosis of chromophobe renal cell carcinoma (non-ccRCC), the next step is to determine the histopathological TNM stage and grade. The provided information indicates a stage of pT3a N0 MX.  Step 2: Determination of Surveillance Regimen Based on the provided guidelines and the TNM stage of pT3a with no lymph node involvement (N0) and no distant metastasis assessed (MX), the patient falls into the high-risk category for recurrence due to the size of the tumour and the invasion into perinephric fat.  For non-ccRCC like chromophobe renal cell carcinoma with pT3a staging, the following surveillance regimen would be appropriate as per the EAU Guidelines on RCC - Surveillance:  First year: CT imaging at 3, 6, and 12 months. Second year: CT imaging every 6 months. Third year: CT imaging every 6 months. Beyond 3 years: Annual CT imaging. Beyond 5 years: CT imaging every two years. Output: Histopathological Interpretation: Chromophobe renal cell carcinoma, AJCC staging pT3a N0 MX, with tumour size > 10 cm and invasion into renal sinus and perinephric fat. High risk of recurrence.  Follow-up Imaging Surveillance Regimen:  First year post-surgery: CT scans at 3, 6, and 12 months. Second and third years post-surgery: CT scans every 6 months. After 3 years post-surgery: Annual CT scans. After 5 years post-surgery: CT scans every two years. These surveillance intervals are designed for oncological follow-up to monitor for any signs of recurrence. |

ccRCC = clear cell renal cell carcinoma; pRCC = papillary renal cell carcinoma; chRCC = chromophobe renal cell carcinoma; Int = intermediate

**Supplementary Figure 3.** Concordance of ChatGPT-determined risk profiles and surveillance regimens to the EAU Guidelines on RCC.


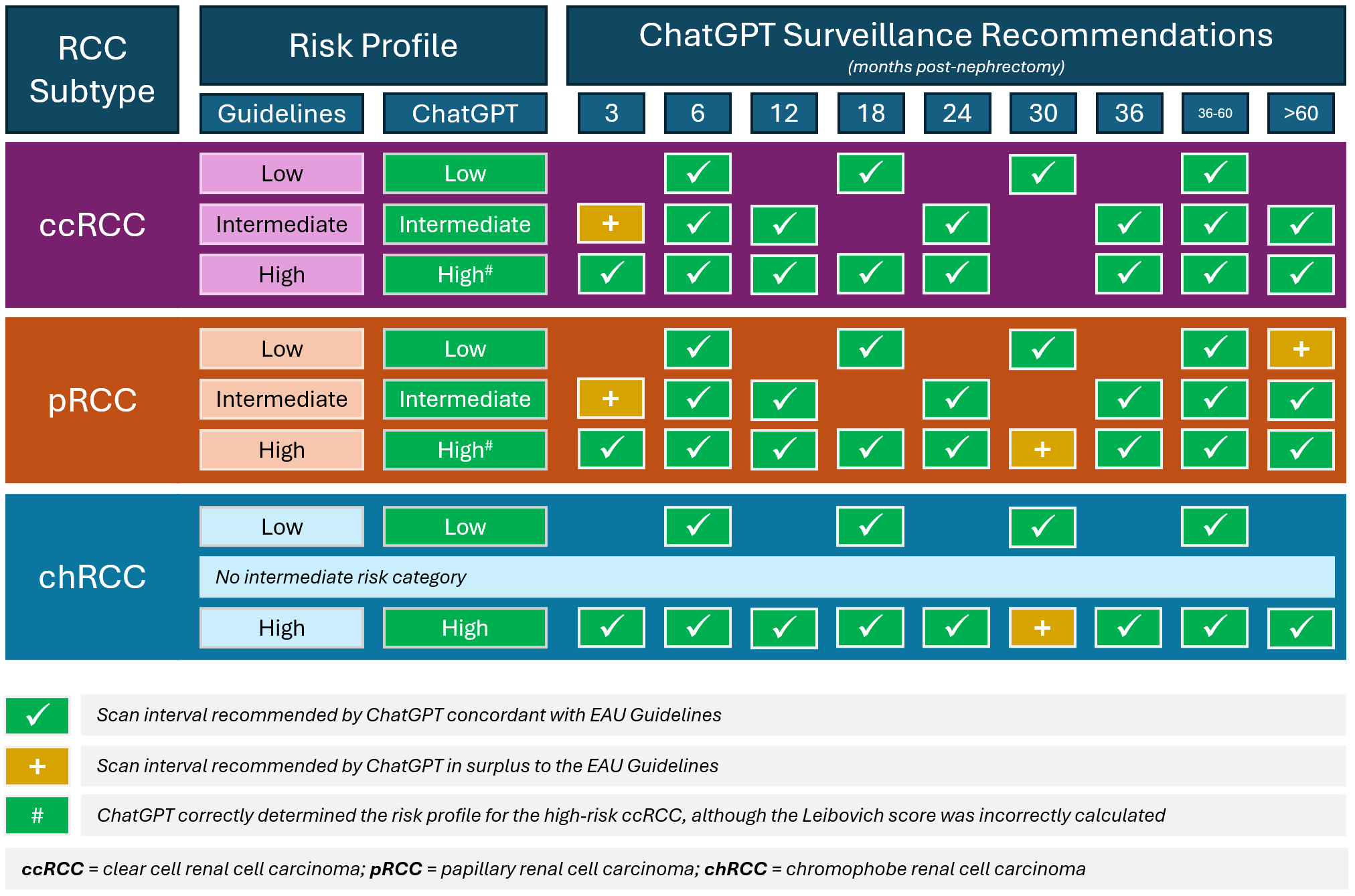

Supplement: Supplementary file 1 — Figure S1. Custom GPT Instructions. The custom GPT is available at https://chat.openai.com/g/g-eIATeIq2o-rcc-surveillance-imaging. Table S2. Input (histopathology report) to custom GPT with respective output. Figure S3. Concordance of ChatGPT‐determined risk profiles and surveillance regimens to the EAU Guidelines on RCC. [file BCO2-6-e427-s001.docx]
